# Identifying Opioid Use Disorder in the Emergency Department: Multi-System Electronic Health Record–Based Computable Phenotype Derivation and Validation Study

**DOI:** 10.2196/15794

**Published:** 2019-10-31

**Authors:** David Chartash, Hyung Paek, James D Dziura, Bill K Ross, Daniel P Nogee, Eric Boccio, Cory Hines, Aaron M Schott, Molly M Jeffery, Mehul D Patel, Timothy F Platts-Mills, Osama Ahmed, Cynthia Brandt, Katherine Couturier, Edward Melnick

**Affiliations:** 1 Yale Center for Medical Informatics Yale University School of Medicine New Haven, CT United States; 2 Information Technology Services Yale New Haven Health New Haven, CT United States; 3 Department of Emergency Medicine Yale University School of Medicine New Haven, CT United States; 4 North Carolina Translational and Clinical Sciences Institute University of North Carolina School of Medicine Chapel Hill, NC United States; 5 Department of Emergency Medicine University of North Carolina School of Medicine Chapel Hill, NC United States; 6 Department of Emergency Medicine Mayo Clinic Rochester, MN United States; 7 Department of Health Sciences Research Mayo Clinic Rochester, MN United States

**Keywords:** electronic health records, emergency medicine, algorithms, phenotype, opioid-related disorders

## Abstract

**Background:**

Deploying accurate computable phenotypes in pragmatic trials requires a trade-off between precise and clinically sensical variable selection. In particular, evaluating the medical encounter to assess a pattern leading to clinically significant impairment or distress indicative of disease is a difficult modeling challenge for the emergency department.

**Objective:**

This study aimed to derive and validate an electronic health record–based computable phenotype to identify emergency department patients with opioid use disorder using physician chart review as a reference standard.

**Methods:**

A two-algorithm computable phenotype was developed and evaluated using structured clinical data across 13 emergency departments in two large health care systems. Algorithm 1 combined clinician and billing codes. Algorithm 2 used chief complaint structured data suggestive of opioid use disorder. To evaluate the algorithms in both internal and external validation phases, two emergency medicine physicians, with a third acting as adjudicator, reviewed a pragmatic sample of 231 charts: 125 internal validation (75 positive and 50 negative), 106 external validation (56 positive and 50 negative).

**Results:**

Cohen kappa, measuring agreement between reviewers, for the internal and external validation cohorts was 0.95 and 0.93, respectively. In the internal validation phase, Algorithm 1 had a positive predictive value (PPV) of 0.96 (95% CI 0.863-0.995) and a negative predictive value (NPV) of 0.98 (95% CI 0.893-0.999), and Algorithm 2 had a PPV of 0.8 (95% CI 0.593-0.932) and an NPV of 1.0 (one-sided 97.5% CI 0.863-1). In the external validation phase, the phenotype had a PPV of 0.95 (95% CI 0.851-0.989) and an NPV of 0.92 (95% CI 0.807-0.978).

**Conclusions:**

This phenotype detected emergency department patients with opioid use disorder with high predictive values and reliability. Its algorithms were transportable across health care systems and have potential value for both clinical and research purposes.

## Introduction

### Background

In the decade since the Health Information Technology for Economic and Clinical Health Act of 2009 was enacted, US hospitals have achieved greater than 96% adoption of electronic health records (EHRs) [1]. EHRs are projected to store 2314 exabytes (1 exabyte=approximately 1 billion GB) of health data by 2020 [2]. This wealth of data has been touted as *a practically inexhaustible source of knowledge to fuel a learning health care system* [3]. Yet at this time, significant challenges remain for using clinical data for research and optimization of health care delivery [4]. Integral to addressing these challenges and studying an intervention in actual clinical care is the ability to accurately and reliably identify patients with particular diagnoses or medical conditions across heterogeneous systems [4-6]. An EHR-based computable phenotype aims to do precisely that. Henceforth, it is referred to as an EHR-based *phenotype*, defined as a set of data elements and logical expressions used to identify individuals or populations (ie, cohorts) with particular diagnoses or medical conditions via clinical characteristics, events, and service patterns that are ascertained using a computerized query of an EHR system or data repository [5,7]. Phenotypes are typically used in clinical trial recruitment to identify cohorts with specific conditions using diverse data sources [5]. They are also increasingly used to define an authoritative standard for electronic clinical quality measure reporting [8].

An estimated 2.1 million people in the United States have opioid use disorder (OUD) [9], and over 33,000 opioid-related deaths occur annually, a number projected to increase to more than 81,000 by 2025 [10,11]. From 2016 to 2017, emergency departments (EDs) experienced a 30% increase in visits for opioid overdose [12]. Buprenorphine, a partial opioid agonist generally combined with an antagonist (naloxone), is an effective treatment for OUD that decreases mortality (from approximately 5% to 3% annually following an ED visit for opioid overdose), withdrawal symptoms, craving, and opioid use [13-15]. Initiating buprenorphine in the ED doubles the rate of addiction treatment engagement in ED patients with OUD [16]. However, ED-initiated buprenorphine has not yet been adopted into routine emergency care [17,18].

### Objectives

Phenotyping could be used as a clinical tool to identify patients likely to benefit from ED-initiated buprenorphine or other interventions and as a research tool to identify patients who should be included in large-scale intervention studies of OUD interventions. We will conduct a multi-system pragmatic trial of user-centered clinical decision support to implement EMergency department-initiated BuprenorphinE for opioid use Disorder (EMBED) across 20 EDs in 5 health care systems [19]. EHR phenotyping will allow pragmatic comparison of the effectiveness of the EMBED intervention to usual care on outcomes in ED patients with OUD in the upcoming EMBED trial (primary outcome—adoption of ED-initiated buprenorphine in routine emergency care). Our objective in this study was to derive and validate an EHR-based computable phenotype to identify ED patients with OUD using structured data; physician validation based on chart review was used as the reference standard. This phenotype will be used to inform patient identification and data collection for the subsequent EMBED pragmatic trial.

## Methods

### Study Setting and Sample

This phenotype was created for the purposes of identifying patients with OUD who could benefit from ED-initiation of buprenorphine in a subsequent trial or quality improvement initiatives. Therefore, the phenotype only included ED patients who were discharged from the hospital (ie, not admitted as inpatients), were not currently prescribed buprenorphine, methadone, or naltrexone as medication treatment for OUD, and were not pregnant (as buprenorphine with naloxone may not be safe for pregnant women and its use requires more expertise than clinical decision support). This study was performed within the XXXX Health System in YYYY and XXXX Health System in YYYY by identifying a cohort of adults (>18 years of age) with ED encounters between November 1, 2017, and October 31, 2018, in the EHR. The 2 health care systems use different billing companies, but the same EHR vendor (Epic; Epic Systems Corporation). Data were extracted from the EHR of each hospital using local Epic Clarity databases (Epic; Epic Systems Corporation). These data comprised information available within the EHR on the date of service of the ED visit in question. Approval for this study was provided by the Institutional Review Boards of the respective institutions (Protocol IDs 2000022749 [internal validation] and 18-2653 [external validation]).

### Clinical Definition of Opioid Use Disorder

Although psychiatric evaluation is the gold standard for diagnosing OUD, within the emergency medicine (EM) context, diagnosis if performed is based on the Diagnostic and Statistical Manual of Mental Disorders, 5th Edition (DSM-5) criteria [20]. The DSM-5 specifies 11 criteria for the diagnosis of OUD, with qualifiers for remission [21]. It specifies that OUD consists of “A problematic pattern of opioid use leading to clinically significant impairment or distress, as manifested by at least two of [...eleven criteria], occurring within a 12-month period.” These criteria include opioids taken repeatedly, continuously, and in larger amounts over a longer period than was intended, resulting in sequelae such as tolerance, withdrawal, craving, desire to cut down, failure to fulfill or engage in social and role obligations (such as at work, school, or home), and continued use despite problems related to use.

### Electronic Health Record Definition

**Figure 1:**
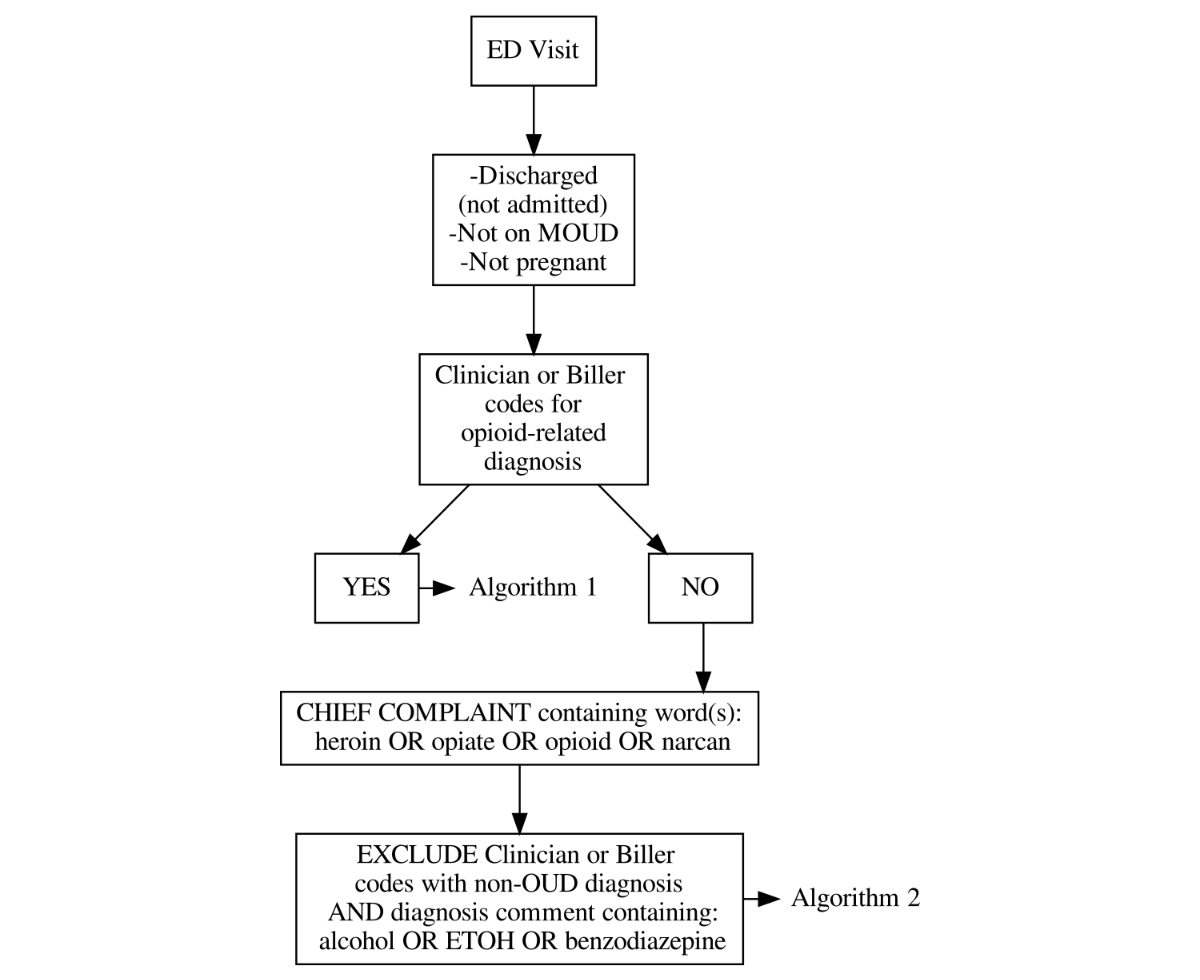
Flow diagram of phenotypes. ED: emergency department; MOUD: medication for opioid use disorder; ETOH: ethyl alcohol; OUD: opioid use disorder.

The computable phenotype algorithm was developed based on data elements from available primary care OUD phenotypes [22,23] with additions and revisions based on the clinical judgment of an EM attending physician and clinical informaticist as well as available and high-yield structured ED data elements as judged by the health system’s medical director of Information Technology (HP). To maximize the yield and performance of the phenotype, 2 separate algorithms were created (Figure 1). Algorithm 1 is a diagnostic coding–based approach to identifying patients with OUD, utilizing opioid-related International Classification of Diseases, Tenth Revision, Clinical Modification (ICD-10) diagnostic codes associated with the ED visit (as coded by a clinician or medical coder, Table 1).

**Table 1:** List of International Classification of Diseases, Tenth Revision (ICD-10) codes for opioid-related diagnoses used for Algorithm 1 case detection.

ICD-10^a^ code	Description
F11	Opioid-related disorders
T40.0	Poisoning by, adverse effect of, and underdosing of opium
T40.1	Poisoning by, adverse effect of, and underdosing of heroin
T40.2	Poisoning by, adverse effect of, and underdosing of other opioids
T40.3	Poisoning by, adverse effect of, and underdosing of methadone
T40.4	Poisoning by, adverse effect of, and underdosing of other synthetic narcotics
T40.6	Poisoning by, adverse effect of, and underdosing of other and unspecified narcotics

^a^ICD-10: International Classification of Diseases, Tenth Revision.

Algorithm 2 identifies patients who have not been captured by Algorithm 1 but have information in their ED chief complaint suggestive of OUD. This algorithm flagged patients if the words *heroin*, *opiate*, *opioid*, or *narcan* were included in their chief complaint for the ED visit. However, as naloxone is often used in ED patients with undifferentiated altered mental status or overdose, patients with *narcan* in their chief complain who did not have an OUD-related final diagnosis were excluded. Upon preliminary testing of the algorithm, the 2 most frequent false-positive diagnoses were alcohol- and benzodiazepine-related. Visits with these chief complaints but alcohol- and benzodiazepine-related final diagnoses were removed by excluding patients with the words *alcohol* or *benzodiazepine* in their final ED diagnosis.

### Structured Query Language Implementation of the Phenotype

After the 2 algorithms were reviewed, finalized, and approved by the investigative team, individual elements of each algorithm were converted into structured query language (SQL). The computable phenotype algorithm was written to be deployed in the Epic EHR systems across both the health systems. Data structures within Epic were mapped to each of the concepts (which were standardized across hospitals in the system) by a clinical informatics expert (HP). This eliminated the necessity of translating concepts to local codes within hospitals. Sample queries were run, and HP verified charts for accuracy. The SQL query and data dictionary (Multimedia Appendix 1, SQL file and data dictionary) were assembled by HP and reviewed for accuracy and comprehensiveness by HP, EM, and DC. The possible values of each variable are described in Figure 1 and expanded upon in Tables 1 and 2. Once all of the elements of the phenotype were codified in SQL, the algorithms were applied to the study population’s ED medical records.

**Table 2:** Algorithm 2 case definition variables.

EHR^a^ data variable	Criteria for suspected opioid use disorder
Chief complaint	Reason for visit contains the words heroin; opiate; opioid Reason for visit comment contains the word narcan
Diagnosis description	Not Algorithm 1 positive, that is, does not contain ICD-10^b^ codes F11 or T40. 0-40.6 listed in Table 1 Does not contain the words alcohol, EtOH, benzodiazepine

^a^EHR: electronic health record.

^b^ICD-10: International Classification of Diseases, Tenth Revision.

### Evaluation Phase 1: Internal Validation

Following the implementation of the computable phenotype algorithm, internal validation was performed using a sample of 125 charts retrieved from the XXXX health system EHR by a clinical informaticist (HP). A total of 75 charts were intended to be representative of the resulting OUD phenotypes with 50 of these charts meeting Algorithm 1 criteria and the other 25 meeting Algorithm 2 criteria. The other 50 charts were *phenotype negative* (ie, not satisfying criteria for either algorithm)**.** Charts were selected at random from the cohort with ED visits from April 10, 2018, to August 1, 2018, across the health systems and reviewed during August 2018 to October 2018 for the internal validation phase and December 2018 to January 2019 for the external validation phase. As the chart reviewers were given access to the patient’s full chart, the time window for the charts was deliberately narrow to avoid postvisit information (eg, of someone who subsequently develops OUD that was not present on the date of the ED visit) confounding the accuracy of the chart review of the ED visit.

### Evaluation Phase 2: External Validation

The external validation cohort was constructed by a clinical informaticist (WKR) with 20,000 randomly sampled ED visits occurring between November 1, 2017, and October 31, 2018 across the XXXXX health system. We picked this number of charts given the rate of phenotype-positive charts in the internal validation cohort with a goal of estimating sensitivity of the phenotype based on prevalence in this random sample. A total of 55 charts met Algorithm 1 criteria. Of those not positive for Algorithm 1, 1 chart met Algorithm 2 criteria. Of the remaining negative cases, a 0.25% (50/200) random sample produced 50 charts for review. Cases positive for Algorithm 1 or 2 were combined owing to the low yield of a single chart identified as Algorithm 2 positive.

### Chart Review

Each chart was reviewed independently and separately by 2 EM physicians (internal validation: DPN, EB; external validation: CH, AMS) blinded to the results and the algorithms and the decision of the other reviewer. All cases of disagreement were adjudicated by a third EM physician reviewer (internal validation: KC; external validation: TFP) also blinded to the results of the algorithms and the decision of the other reviewers. Reviewers were asked to diagnose patients as *OUD-positive* or *OUD-negative* based upon a review of EHR data available up to and on the date of the ED visit (but not after the ED visit), their clinical judgment, and the DSM-5 OUD diagnostic criteria which were presented to them with each case at the time of review [21]. For cases that were categorized as *OUD-positive*, reviewers were then prompted to select at least 2 of the 11 DSM-5 criteria that informed their diagnosis.

### Analysis

Phenotype performance was assessed using descriptive statistics. A standard 2×2 confusion matrix [24] was configured for analysis of the performance of each algorithm in each phase. The reference standard was the adjudicated diagnosis, whereas the test was the phenotype result. For Algorithm 1 in the internal validation phase (Table 3), the top row included the 50 phenotype-positive charts, and the bottom row included the 50 phenotype-negative charts. For Algorithm 2 in the internal validation phase (Table 3), the top row included the 25 phenotype-positive charts, and the bottom row included the 25 phenotype-negative charts. In the external validation phase, the algorithms were combined because of low incidence of Algorithm 2-positive (Table 3), with 56 positive and 50 negative. Interrater reliability was reported using Cohen kappa. Analyses were conducted with the scikit-learn package (version 0.19.2) in Python (version 2.7.12) for internal validation and Stata (StataCorp, version 14) for external validation.

**Table 3:** Confusion matrices for validation phases (disease present: reference standard).

Test		Result			
		Reviewers +	Reviewers −	Predictive value	95% CI
**Algorithm 1 (internal validation)**					
	Phenotype +	48	2	0.96^a^	0.863-0.995
	Phenotype −	1	49	0.98^b^	0.893-0.999
**Algorithm 2 (internal validation)**					
	Phenotype +	20	5	0.8^a^	0.593-0.932
	Phenotype −	0	25	1.0^b^	0.863-1.000^c^
**Combined phenotype (external validation)**					
	Phenotype +	53	3	0.95^a^	0.851-0.989
	Phenotype −	4	46	0.92^b^	0.807-0.978

^a^Positive predictive value.

^b^Negative predictive value.

^c^97.5%, one-sided.

## Results

Among ED visits resulting in discharge from November 1, 2017, to October 31, 2018, across the 13 EDs in the 2 health care systems, a total of 474,176 unique ED visits (discharged patients only) with an average of 36,475 ED visits per year per site were identified. A total of 2294 of these visits were phenotype-positive with an average of 176 (median 104) phenotype-positive visits per site. Site visit by volume is presented in Table 4.

**Table 4:** Annual volume of emergency department (ED) visits meeting phenotype criteria (November 1, 2017, to October 31, 2018, ED discharges only).

Validation			Total patients (n)	Total visits (n)	Algorithm 1 (n)	Algorithm 2 (n)
**Internal**						
	**Department**					
		Hospital X I	44,291	67,995	343	49
		Health System X II	22,344	29,309	56	11
		Health System X III	24,738	38,128	251	46
		Health System X IV	27,220	44,505	324	73
		Health System X V	44,780	65,837	509	70
		Health System X VI	17,797	22,540	25	0
	Total		181,170	268,314	1508	249
	Average		30,195	44,719	251.3	41.5
**External**						
	**Department**					
		Health System Y I	9818	15,749	37	4
		Health System Y II	15,220	25,556	91	2
		Health System Y III	22,332	30,912	57	4
		Health System Y IV	22,080	38,086	100	4
		Health System Y V	5467	6190	24	1
		Health System Y VI	34,576	46,335	98	0
		Health System Y VII	32,879	43,034	110	5
	Total		142,372	205,862	517	20
	Average		20,339	29,409	74	3

### Internal Validation Cohort

In the internal validation cohort of 125 charts, reviewers disagreed on the classification of 3 charts (agreement=97%; kappa=0.95), with the adjudicator identifying the 2 discordant Algorithm 1 cases as not having OUD and the 1 discordant Algorithm 2 case as having OUD. Algorithm 1 had a positive predictive value (PPV) of 0.96 (95% CI 0.863-0.995) and a negative predictive value (NPV) of 0.98 (95% CI 0.893-0.999; Table 3). Algorithm 2 had a PPV of 0.8 (95% CI 0.593-0.932) and an NPV of 1.0 (one-sided 97.5% CI 0.863-1; Table 3). The most frequently met current DSM-5 criteria were “opioids taken in larger amounts or over a longer period than was intended” or “recurrent use in situations in which it is physically hazardous,” whereas the least frequent criteria were those describing social dysfunction related to the use of opioids (such as “recurrent opioid use resulting in a failure to fulfill major role obligations at work, school, or home” or “important social, occupational, or recreational activities are given up or reduced because of opioid use”).

### External Validation Cohort

In the external validation cohort of 106 charts, reviewers disagreed on the classification of 8 charts (agreement=92.5%; kappa=0.85). A total of 3 of the 8 discordant cases were phenotype-positive, of which the adjudicator determined 2 as having OUD. Of the 5 discordant cases that were phenotype-negative, the adjudicator identified 3 as having OUD. The combined phenotype had a PPV of 0.95 (95% CI 0.851-0.989) and an NPV of 0.92 (95% CI 0.807-0.978; Table 3). The most frequently met current DSM-5 criteria were “opioids are often taken in larger amounts or over a longer period than was intended” and “craving, or a strong desire or urge to use opioids,” whereas the least frequent criterion was “important social, occupational, or recreational activities are given up or reduced because of opioid use.”

## Discussion

### Principal Findings

With an externally validated PPV of 0.95 and NPV of 0.92, the combined phenotype derived and validated for this study performed remarkably well in predicting OUD in ED patients across 2 large health care systems. The strength of the phenotype’s classification performance may be because of the possibility that the algorithm and the reviewers were using similar (if not the same) information from patients’ charts.

In both the internal and external validation chart reviews, the most common DSM criterion selected by the reviewers was “opioids are often taken in larger amounts or over a longer period than was intended.” In the internal validation phase, the second most common criterion was “recurrent opioid use in situations in which it is physically hazardous,” whereas the second most common criterion in the external validation phase was “craving, or a strong desire or urge to use opioids.” Although desire and effort to cut down or control opioid use are specific diagnostic criteria, they were inconsistently applied by the reviewers. As these specific criteria are not explicitly documented in the routine emergency care, the reviewers instead had to infer which criteria to apply to cases using available documentation. In both chart review phases, the least frequently identified criteria were those describing failures in social behavior as they pertained to the use of opioids. This could be because of the fact that ED billing requirements do not require detailed documentation of social history, and the impact of opioids on social behaviors usually has limited value for assisting clinicians in making a diagnosis during emergency care [25].

Given the limitations of ED documentation, our phenotype benefited from incorporation of available structured data elements from the data dictionaries created in previous work to develop EHR phenotypes for primary care patients on chronic opioid therapy at risk for problematic opioid use [22,23]. Given the difference in populations and objectives between this study and the primary care OUD phenotype, it is difficult to compare the differences in their phenotypes’ performance. In particular, the previous work focused on the performance of natural language processing for identifying risk for problematic opioid use in patients for whom differences in the signs and symptoms of OUD might be more nuanced: every patient included in that study was on chronic opioid therapy. The goal of that study was to capture the presence of OUD symptoms using free-text notes—a complex machine learning problem. In contrast, our study included a broader population (all patients presenting to the ED were eligible for inclusion in the phenotype-negative sample), and our phenotype drew on structured data elements including diagnoses and chief complaints; these structured data generally reflect the clinical judgment of people who have directly observed the patient and determined that OUD was likely.

A strength of our study compared with previous EHR phenotype work is the external validation of the phenotype’s performance via chart review in the second health care system. For example, the HIV EHR phenotype developed by Paul et al [26] performs well, but its transportability and performance in outside health care systems are not known [27]. External validation is particularly important for EHR phenotypes that rely on documentation and diagnostic codes as documentation and diagnostic codes are dependent on local practice patterns by clinicians and coders, both of which could vary within and across health care systems.

The phenotype described here will be used as part of a subsequent pragmatic trial to be deployed across multiple health care systems to identify patients who may have been candidates for ED-initiated buprenorphine—these cases will form the denominator of a measure to assess what proportion of those potentially eligible actually received buprenorphine. As most patients evaluated using the phenotype will screen negative, our approach would likely result in a high number of false negatives in a true epidemiologic evaluation. As we were trying to maximize specificity for a pragmatic trial, the phenotype’s classification performance will meet the trial’s needs to screen patients for eligibility for ED-initiated buprenorphine with high specificity. Furthermore, the goal was not to definitively determine a diagnosis of OUD for each patient. For clinical practice, any patient identified as having OUD by this phenotype would require confirmation using an in-person assessment. As the capacity and expectation of EDs to treat OUD expands, so also does the value of an accurate EHR phenotype that could be used to identify patients who might benefit from treatment including ED-initiated buprenorphine and referral for ongoing medication treatment for OUD.

### Limitations

The primary limitation of this study is the use of retrospective ED chart review as a reference standard for the diagnosis of OUD. Our chart review process was robust and included all clinical documentation up to date of the ED visit. However, a full diagnostic assessment by a psychiatrist or addiction medicine specialist would be the gold standard to establish a diagnosis of OUD. If available, it is possible that such an assessment would differ from chart review alone.

External validation in an outside health care system strengthens the evidence for the generalizability of our phenotype. The external system uses the same EHR vendor but a different billing and coding company. It is unknown how the EHR phenotype would perform in systems using other EHR platforms. In addition, transportability issues have been discovered in preliminary estimates from a third health system because of differences in structured data capture of the chief complaint. In the external validation phase, the second algorithm did not identify a substantial number of cases. This suggests that there are likely local practice patterns in documentation or coding that may have affected the transportability of this EHR phenotype [27]. Although local phenotype development and adaptation could overcome this limitation, the overall classification performance remained strong in the external validation phase. Furthermore, in the internal validation phase, the individual algorithms maintained high PPV values (0.96 and 0.8, respectively) and NPV values (0.98 and 1.0).

In future work, the efficacy of the phenotype algorithms will be tested in the EMBED trial, and the question as to whether these algorithms can function in a pragmatic ED setting will be answered. Statistically, the cohort selection and chart review performed here did not obtain a patient population reflective of the true prevalence of the disease, and as such, sensitivity and specificity calculations would not provide an accurate reflection of the phenotype’s performance in a true ED population. Assessment of sensitivity for events with low base rates is inherently unreliable. A very large sample size would be necessary for a precise estimate of sensitivity. Therefore, the assessment of sensitivity in this analysis is limited. To address this limitation, we report only PPV and NPV here. Estimating sensitivity and specificity for the external validation study can be done by inflating the phenotype-negative row numbers in the external validation confusion matrix by the sampling factor (Table 3) to represent 19,944 patients who screened negative. The sampling factor of approximately 399 (19,944/50) would change this row to extrapolated values of 1596 (false negatives) and 18,354 (true negatives). These extrapolated values would yield a sensitivity of 3.2% with an extremely wide confidence interval and specificity of 99.9%. This wide range could be explained by the false negatives that the phenotype is not identifying. Given the current opioid crisis, we know that the rates of OUD are high, and there are likely many individuals with occult OUD that is not being identified in the ED as they may be presenting with medical complaints unrelated to their OUD comorbidity. For example, abdominal pain and chest pain are the 2 most common presenting complaints to EDs nationally. There is likely a large population of ED patients with these complaints that have OUD that goes unrecognized in the ED. Future work should screen for more precise estimates of undiagnosed OUD in the ED population.

In the upcoming trial, further evaluation of the phenotype algorithms’ performance will begin to address the intra- and intersite population sensitivity and specificity. To further refine the algorithm’s ability to discern between true and false positives, logistic regression is planned to predict future OUD-coded diagnoses given information from previous visits, such that variables can be removed given their performance in the regression model. Future work will also attempt to quantify the rate of false negatives through extended manual review and to determine whether changes to the algorithm improve sensitivity. The long-term goal of future work is to standardize the representation of the algorithms such that they can be portable beyond Epic to other EHR vendors as well as explore additional information retrieval techniques [28]. A more comprehensive validation could establish more reliable sensitivity estimates by use of a gold standard estimate of true prevalence of OUD in the ED population by screening a representative ED population for OUD with DSM-5 diagnostic criteria [21].

### Conclusions

An EHR phenotype derived and internally and externally validated for the purposes of a pragmatic trial to test the effectiveness of user-centered clinical decision support to increase the adoption of ED-initiated buprenorphine performed reliably and accurately to identify ED patients with OUD. The 2 algorithms comprising the phenotype were transportable across health care systems and have potential value for both clinical quality improvement interventions as well as research endeavors. Standardization of the phenotype will support efforts to use clinical phenotyping as an evidence-based tool at the front line of clinical practice.

## Appendix

Multimedia Appendix 1 SQL code to retrieve records from Epic Clarity database.

## Abbreviations

DSM-5: Diagnostic and Statistical Manual of Mental Disorders, 5th Edition
ED: emergency department
EHR: electronic health record
EM: emergency medicine
EMBED: EMergency department-initiated BuprenorphinE for opioid use Disorder
ICD-10: International Classification of Diseases, Tenth Revision
NIH: National Institutes of Health
NPV: negative predictive value
OUD: opioid use disorder
PPV: positive predictive value
SQL: structured query language
